# Two New Chemical Constituents from the Stem Bark of *Garcinia mangostana*

**DOI:** 10.3390/molecules19067308

**Published:** 2014-06-04

**Authors:** Irene See, Gwendoline Cheng Lian Ee, Soek Sin Teh, Arifah Abdul Kadir, Shaari Daud

**Affiliations:** 1 Department of Chemistry, Faculty of Science, Universiti Putra Malaysia, 43400 Serdang, Selangor, Malaysia; 2 Department of Veterinary Pre-Clinical Science, Faculty of Veterinary Medicine, Universiti Putra Malaysia, 43400 Serdang, Selangor, Malaysia; 3 Department of Chemistry, Faculty of Applied Sciences, Universiti Teknologi MARA, 26400 Jengka, Pahang, Malaysia

**Keywords:** *Garcinia mangostana*, Clusiaceae, prenylated xanthone, benzophenone

## Abstract

A detailed chemical study on the ethyl acetate and methanol extracts of the stem bark of *Garcinia mangostana* resulted in the successful isolation of one new prenylated xanthone, mangaxanthone B (**1**), one new benzophenone, mangaphenone (**2**), and two known xanthones, mangostanin (**3**) and mangostenol (**4**). The structures of these compounds were elucidated through analysis of their spectroscopic data obtained using 1D and 2D NMR and MS techniques.

## 1. Introduction

The Clusiaceae family consists of approximately 40 genera [1], including *Garcinia*, *Mesua* and *Cratoxylum* [2]. Some Clusiaceae plants are used in traditional medicine to treat various illnesses. For example, *Garcinia schomburgkianaPierre* is used to treat coughs and menstrual problems [3] while *Mesua ferrea* is used in the treatment for dyspepsis and renal disease [4]. Clusiaceae plants contain numerous biologically active secondary metabolites, such as benzophenones, xanthones, coumarins and flavonoids. The pharmacological properties of these secondary metabolites include antifungal activity in *Calophyllum thwaitesii* [5], antioxidant activity in *Cratoxylum cochinchinense* [6], antimicrobial and antibacterial activities in *Garcinia cowa* [7] and anticancer activity in *Garcinia paucinervis* [8]. *Garcinia* plants are mainly found in tropical countries such as Malaysia, Thailand and Brazil [1]. *Garcinia* plants are currently being more avidly studied due to their abilities to treat dysentery, pain, tapeworm infestations and many more ailments [9]. Being a member of the *Garcinia* genus, the mangosteen is also well known to be a rich source of xanthones and benzophenones, especially polyprenylated xanthones and oxygenated xanthones [10]. These secondary metabolites have been reported to possess biological properties against fungi [11], bacteria [12,13] and also cancer [14,15]. Ryu and co-workers also reported that some oxygenated xanthones from the seedcases of this plant possess neuraminidase inhibitory activity [16]. The discoveries of these beneficial secondary metabolites have revitalized our interest to investigate more extensively on the stem bark of *Garcinia mangostana*. Herein, we describe the isolation as well as the characterization of a new prenylated xanthone, mangaxanthone B (**1**) and a new benzophenone, mangaphenone (**2**), along with two other known xanthones mangostanin (**3**) and mangostenol (**4**).

## 2. Results and Discussion

**Figure 1:**
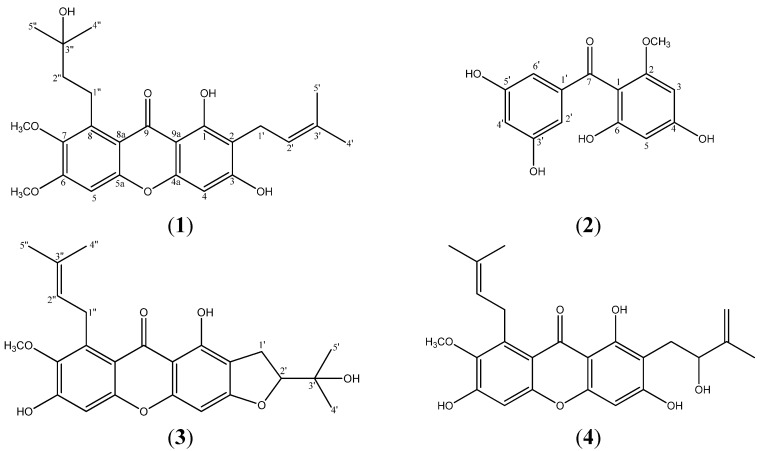
Structures of compounds **1**–**4**.

The stem bark of *Garcinia mangostana* was extracted with ethyl acetate (EtOAc) and methanol (MeOH) followed by fractionation of these extracts to obtain a new prenylated xanthone, mangaxanthone B (**1**), a new benzophenone, mangaphenone (**2**) and two other known compounds mangostanin (**3**) and mangostenol (**4**). Structural elucidation of these compounds were performed by analysing their spectroscopic data. The structures of compounds **3** and **4** were confirmed by comparing their spectroscopic data with data available from the literature. The structures of compounds **1**–**4** are illustrated in Figure 1.

Compound **1** was isolated as a yellow solid (m.p. = 194–195 °C) and found to have a molecular formula of C_25_H_30_O_7_ through the EIMS spectrum, which showed a molecular ion peak at *m*/*z* 442. The FTIR absorption indicated the existence of OH (3447 cm^−1^), alkane side chain CH (2938 cm^−1^), aromatic moiety C=C (1457 cm^−1^), CO (1601 cm^−1^) and alkene moiety CH (826 cm^−1^) bands. Besides, the λ_max_ at 209, 245, 262, 316 and 353 nm in the UV-Visible spectrum are the characteristic absorption bands of an aromatic benzene chromophore, which indicated the presence of a xanthone nucleus.

In the ^1^H-NMR spectrum, signals at *δ*_H_ 6.38 (s, 1H, H-4) and 6.90 (s, 1H, H-5) imply the presence of a xanthone nucleus. On the other hand, the prenyl moiety was evident from proton resonances at *δ*_H_ 3.32 (*br* d, 2H, H-1'), 5.25 (t, 1H, *J* = 6.9 Hz, H-2'), 1.76 (s, 3H, H-4') and 1.62 (s, 3H, H-5') while the characteristic proton resonances of the 3-hydroxy-3-methylbutyl moiety were seen at *δ*_H_ 3.42 (m, 2H, H-1''), 1.70 (m, 2H, H-2'') and 1.28 (s, 6H, H-4'', H-5''). The ^1^H-NMR spectrum also showed characteristic resonances of a chelated hydroxyl group at *δ*_H_ 13.74 (s, 1H, 1-OH) and two methoxyl groups at *δ*_H_ 4.00 (s, 3H, 6-OCH_3_) and 3.79 (s, 3H, 7-OCH_3_) (see Table 1).

**Table 1:** ^1^H-NMR (500 MHz) and ^13^C-NMR (125 MHz) spectroscopic data for compound **1** (in Me_2_CO-*d*_6_) and **2** (in CD_3_OD).

Position	1		2	
	*δ*_H_	*δ*_C_	*δ*_H_	*δ*_C_
1		160.8		106.4
2		110.3		161.5
3		162.2	5.96 (s)	91.1
4	6.38 (s)	92.3		163.3
4a		154.9		
5	6.90 (s)	98.5	5.96 (s)	95.3
5a		155.4		
6		158.6		163.3
7		144.1		198.4
8		138.4		
8a		111.4		
9		182.0		
9a		102.9		
1'	3.32 ( *br* d)	21.2		142.7
2'	5.25 (t, *J* = 6.3 Hz)	122.6	6.52 (d, *J* = 2.3Hz)	106.5
3'		130.6		158.0
4''	1.76 (s)	17.1	6.39 (t, *J* = 2.3 Hz)	105.7
5'	1.62 (s)	25.1		158.0
6'			6.52 (d, *J* = 2.3 Hz)	106.5
1''	3.42 (m)	22.2		
2''	1.70 (m)	44.9		
3''		69.7		
4'' & 5''	1.28 (s)	28.5		
1-OH	13.74 (s)			
2-OCH_3_			3.54 (s)	54.6
6-OCH_3_	4.00 (s)	55.8		
7-OCH_3_	3.79 (s)	60.4		

Meanwhile, the DEPT experiment indicated that this compound is composed of three methine (*δ*_C_ 92.3, 98.5 and 122.6), three methylene (*δ*_C_ 21.2, 22.2 and 44.9), four methyl (*δ*_C_ 17.1, 25.1, 28.5 × 2), two methoxyl (*δ*_C_ 55.8 and 60.4) and 13 quaternary carbons (*δ*_C_ 69.7, 102.9, 110.3, 111.4, 130.6, 138.4, 144.1, 154.9, 155.4, 158.6, 160.8, 162.2 and 182.0). These results are consistent with the ^13^C-NMR spectrum, which indicated the presence of 25 carbons. The presence of a xanthone skeleton was again obvious in the ^13^C-NMR spectrum, with the signal at *δ*_C_ 182.0, a characteristic signal for the carbonyl group in the xanthone skeleton. Moreover, six oxygenated aromatic carbons [C-7 (*δ*_C_ 144.1), C-4a (*δ*_C_ 154.9), C-5a (*δ*_C_ 155.4), C-6 (*δ*_C_ 158.6), C-1 (*δ*_C_ 160.8) and C-3 (*δ*_C_ 162.2)] were observed in compound **1** after further examination of the DEPT spectrum.

**Figure 2:**
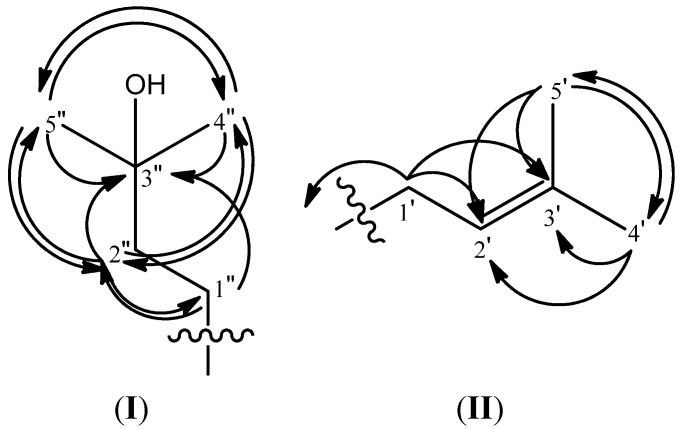
The HMBC correlation of 3-hydroxy-3-methylbutyl (**I**) and prenyl (**II**) moieties in compound **1**.

The HMBC long range ^3^*J* correlations between the chelated hydroxyl group with C-9a (*δ*_C_ 102.9) and C-2 (*δ*_C_ 110.3) and a ^2^*J* correlation with C-1 (*δ*_C_ 160.8) were observed. This allows the assignment of the hydroxyl group to C-1 [*δ*_H_ 13.74 (s, 1H, 1-OH)]. The two prenyl moiety methyl groups resonating at *δ*_H_ 1.76 and 1.62 are correlated to the neighbouring C-3' (*δ*_C_ 130.6) and C-2' (*δ*_C_ 122.6) in the HMBC experiment. Moreover, the linkages between H-1' [*δ*_H_ 3.32 (*br* d, 2H)] and C-2' (*δ*_C_ 122.6) as well as C-3' (*δ*_C_ 130.6) are seen. The linkages between H-2' [*δ*_H_ 5.25 (t, 1H, *J* = 6.3 Hz)] and C-4' (*δ*_C_ 17.1) and C-5' (*δ*_C_ 25.1) are also clearly seen in the experiment (Figure 2). These correlations provide evidence for the presence of the prenyl side chain while the ^2^*J* correlation between H-1' [*δ*_H_ 3.32 (*br* d, 2H)] with C-2 (*δ*_C_ 110.3) and the ^3^*J* correlations of H-1' [*δ*_H_ 3.32 (*br* d, 2H)] with C-3 (*δ*_C_ 162.2) indicated that the prenyl moiety is positioned at C-2. In the COSY analysis, the coupling of H-1' and H-2' was very strong.

**Figure 3:**
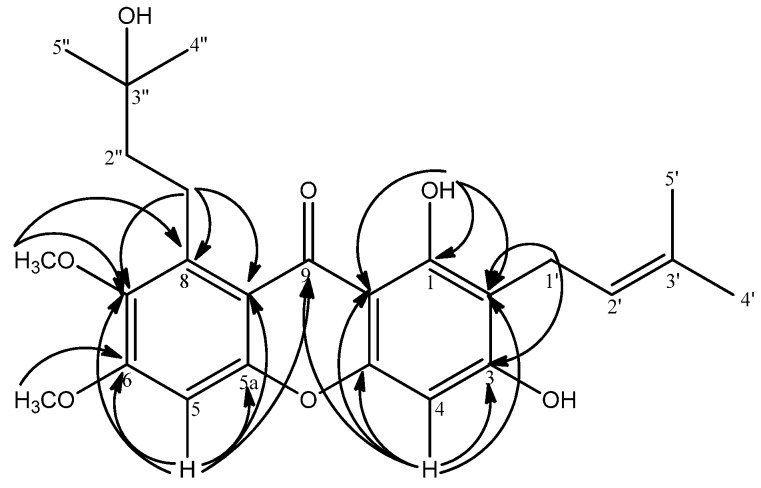
Key HMBC correlations between ^1^H and ^13^C in compound **1**.

A pair of overlapping aliphatic methyls [*δ*_H_ 1.28 (s, 6H, H-4'', H'')] gave clear correlations with C-2'' (*δ*_C_ 44.9) and C-3'' (*δ*_C_ 69.7) indicating the methyls to be attached to C-3''. H-2'' [*δ*_H_ 1.70 (m, 2H)] gave cross peaks to C-4''(*δ*_C_ 28.5), C-5'' (*δ*_C_ 28.5), C-3'' (*δ*_C_ 69.7) and C-1'' (*δ*_C_ 22.2). Meanwhile H-1'' [*δ*_H_ 3.42 (m, 2H)] showed a ^2^*J* correlation with C-2'' (*δ*_C_ 44.9) and a ^3^*J* correlation with C-3'' (*δ*_C_ 69.7). These long range correlation signals allow us to conclude a 3-hydroxy-3-methylbutyl moiety (Figure 2). The coupling of H-1'' and H-2'' was seen in the COSY experiment. Moreover, the cross peaks of H-1'' [*δ*_H_ 3.42 (m, 2H)] to C-7 (*δ*_C_ 144.1), C-8 (*δ*_C_ 138.4), C-8a (*δ*_C_ 111.4) and the cross-peak of H-2'' [*δ*_H_ 1.70 (m, 2H)] to C-8 (*δ*_C_ 138.4) in the HMBC analysis were suggestive of the 3-hydroxy-3-methylbutyl moiety location at C-8. In addition, the two aromatic methoxyl groups [6-OCH3 (δH 4.00) and 7-OCH3 (δH 3.79)] are assigned at C-6 and C-7 because of their ^3^*J* correlations with *δ*_C_ 158.6 (C-6) and *δ*_C_ 144.1 (C-7). The two remaining aromatic methine protons of the xanthone skeleton were determined to be at the two remaining available carbons, C-4 and C-5. The long range correlations between H-4 (*δ*_H_ 6.38) and C-2 (*δ*_C_ 110.3), C-3 (*δ*_C_ 162.2), C-4a (*δ*_C_ 154.9), C-9 (*δ*_C_ 182.0) and C-9a (*δ*_C_102.9) places the *δ*_H_ 6.38 (H-4) singlet at C-4. The signal at *δ*_H_ 6.90 (H-5) was assigned to C-5 based on its HMBC correlations with C-5a (*δ*_C_ 155.4), C-6 (*δ*_C_ 158.6), C-7 (*δ*_C_ 144.1), C-8 (*δ*_C_ 138.4), C-8a (*δ*_C_ 111.4) and C-9 (*δ*_C_ 182.0). Compound **1** differs from a closely related compound, garcinone D [17] in the presence of a methoxyl group at C-6 where garcinone D has a hydroxyl group at that position. The methoxyl group in compound **1** was assigned from the observation of a HMBC correlation between 6-OCH_3_ (*δ*_H_ 4.00) and C-6 (*δ*_C_ 158.6). The HMBC correlations are illustrated in Figure 3. Therefore, compound **1** was elucidated to be 1,3-dihydroxy-8-(3-hydroxy-3-methylbutyl)-6, 7-dimethoxy-2-(3-methyl-2-buten-1-yl)-xanthone and it was named trivially as mangaxanthone B.

Compound **2** was isolated as yellow-brown crystals (m.p. = 245–246 °C) and was found to have a molecular formula of C_14_H_12_O_6_ via the EIMS (*m*/*z* 276 [M^+^]) analysis. The FTIR spectrum exhibited a strong absorption at 1728 cm^−1^, which suggested the presence of a carbonyl group, and another strong absorption at 2924 cm^−1^ representing the aromatic C-H stretching band. An aromatic benzene chromophore which is of a benzophenone skeleton, was confirmed by the maximum absorption peaks of 211, 214 and 309 nm in the UV-Visible spectrum.

The ^1^H-NMR spectrum of compound **2** showed five aromatic proton signals which resonated at *δ*_H_ 5.96 (s, 2H, H-3 and H-5), *δ*_H_ 6.52 (d, 2H, *J* = 2.3 Hz, H-2' and H-6') and *δ*_H_ 6.39 (t, 1H, *J* = 2.3 Hz, H-4'). After a detailed inspection on the ^13^C NMR and DEPT spectra, it was found that the signals in the ^13^C-NMR spectrum indicated 14 carbons which consisted of one methoxyl (*δ*_C_ 54.6), five methine (*δ*_C_ 91.1, 95.3, 105.7 and 106.5 × 2) and eight quaternary carbons (*δ*_C_ 106.4, 142.7, 158.0 × 2,161.5, 163.3 × 2 and 198.4). The benzophenone skeleton of compound **2** was demonstrated by signals resonating at *δ*_C_ 198.4 for the carbonyl group, as well as signals at *δ*_C_ 158.0, 161.5 and 163.3 for the hydroxylated aromatic carbons (see Table 1).

**Figure 4:**
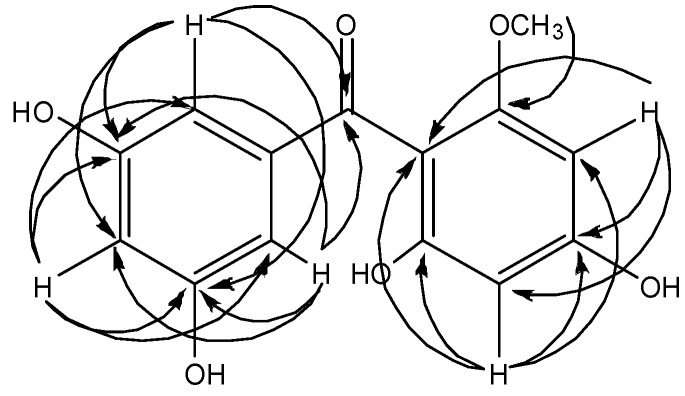
^2^*J*, ^3^*J* and ^4^*J* HMBC correlations between protons and carbons in compound **2**.

The structure of compound **2** was deduced based on the HMBC spectrum (See Figure 4). The aromatic protons at H-2' and H-6' [*δ*_H_ 6.52 (d, 2H, *J* = 2.3 Hz)] showed three-bond connectivities with, C-4' (*δ*_C_ 105.7), and C-7 (*δ*_C_ 198.4). Protons H-2' and H-6' also gave two-bond connectivities with C-3' and C-5' (*δ*_C_ 158.0 × 2) respectively. Meanwhile H-4' has ^2^*J* correlations with C-3' and C-5' (*δ*_C_ 158.0 × 2) and ^3^*J* correlations with C-2' and C-6' (*δ*_C_ 106.5 × 2). The aromatic proton of H-3 [*δ*_H_ 5.96 (s, 1H)] exhibited three-bond (^3^*J*) and two-bond (^2^*J*) connectivities with C-1 (*δ*_C_ 106.4) (^3^*J*), C-5 (*δ*_C_ 95.3) (^3^*J*) and C-4 (*δ*_C_ 163.3) (^2^*J*) respectively. H-5 [*δ*_H_ 5.96 (s, 1H)] also showed correlations with C-1 (*δ*_C_ 106.4) (^3^*J*), C-3 (*δ*_C_ 91.1) (^3^*J*), C-4 and C-6 (*δ*_C_ 163.3 × 2) (^2^*J*) in the HMBC spectrum. Moreover, the methoxyl group was assigned to C-2 because of the ^3^*J* HMBC correlation of the methoxyl proton [*δ*_H_ 3.54 (s, 3H, 2-OCH_3_)] with C-2 (*δ*_C_ 161.5). The observation of a ^2^*J* correlation between H-3 and C-4 implies position 3 to be occupied by H-3. Moreover, we also observed a ^2^*J* correlation between H-5 and C-6 as well as between H-5 and C-4. Therefore, another proton is situated at C-5. 4-OH and 6-OH carbons have the same chemical shift values, thus justifying that the molecule is symmetrical. Hence, the two OH groups are at C-6 and C-4 and not at C-2. Hence, the structure of this compound was elucidated as (4,6-dihydroxy-2-methoxyphenyl)-(3,5-dihydroxyphenyl)methanone and it was given the trivial name mangaphenone.

## 3. Experimental

### 3.1. Plant Material

The stem bark of *Garcinia mangostana* was collected from Melaka, Malaysia. A herbarium specimen (RG221) was deposited at the Herbarium in the Biology Department of UPM.

### 3.2. General

The 1D (^1^H, ^13^C, DEPT) and 2D (COSY, HMQC and HMBC) NMR spectra were recorded on a Unity INOVA 500 MHz NMR instrument using tetramethylsilane (TMS) as the internal standard. EIMS spectra were obtained using a Shimadzu GCMS model QP2010 Plus spectrophotometer. The ultraviolet spectra were recorded on a Shimadzu UV-160A UV-Visible Recording Spectrophotometer. Infrared spectra were obtained using the universal attenuated total reflection (UATR) technique on a Perkin-Elmer 100 Series FT-IR spectrometer. Melting points were measured through Leica Galen III microscope which was equipped with Testo 720 temperature recorder.

### 3.3. Extraction and Isolation

The air-dried powdered stem bark of *Garcinia mangostana* (2.0 kg) was first de-fatted using hexane followed by extraction with ethyl acetate (EtOAc, 3 × 5 L) for 72 h at room temperature then with 70% methanol (MeOH, 3 × 5 L) for another 72 h. The three extracts were concentrated to give 14.22 g of dark brown residue of EtOAc extract and 278.96 g of dark brown residue of MeOH extract. The EtOAc extract was subjected to vacuum column chromatography by eluting with a stepwise gradient system of hexane, chloroform (CHCl_3_), ethyl acetate and methanol to afford 6 fractions. The fourth fraction was further fractionated through column chromatography using hexane–CHCl_3_ and CHCl_3_–MeOH to give 8 fractions. The last fraction was subjected to repeated chromatography by eluting with hexane–EtOAc (7:3) and CHCl_3_–MeOH (9.8:0.2) to furnish compounds **1**, **2** and **3**. Meanwhile, the dry MeOH extract (278.96 g) was suspended in H_2_O and then partitioned with *n*-butanol (*n*-BuOH, 400 mL). The *n*-BuOH soluble portion (2.56 g) was chromatographed in a polarity gradient manner (hexane, hexane–CHCl_3_, CHCl_3_, CHCl_3_–EtOAc, EtOAc–MeOH and MeOH) and afforded eight fractions. Fraction 2 was further purified through column chromatography by using CHCl_3_–MeOH (9:1) and compound **4** was thus obtained.

### 3.4. Spectral Data

*Mangaxanthone B* (**1**). Yellow crystals; m.p. 194–195 °C; UV (EtOH) *λ*_max_ (log ε): 209 (4.40), 245 (4.44), 262 (4.42), 316 (4.29) and 353 (3.79) nm; IR *ν* (cm^−1^): 3447, 3249, 2938, 1601, 1457, 1277 and 826; ^1^H-NMR (500 MHz, Me_2_CO-*d*_6_) and ^13^C-NMR (125 MHZ, Me_2_CO-*d*_6_), see Table 1; EIMS *m*/*z* (rel. int.): 442(32), 424(28), 381(74), 369(43), 368(33), 354(23), 353(100), 327(53) and 325(33).

*Mangaphenone* (**2**). Brownish-yellow crystals; m.p. 245–246 °C; UV (EtOH) *λ*_max_ (log ε): 211 (4.11), 214 (4.12) and 309 (3.77) nm; IR *ν* (cm^−1^): 3599, 2924, 1728, 1261, 804 and 730; ^1^H-NMR (500 MHz, CD_3_OD) and ^13^C-NMR (125 MHZ, CD_3_OD), see Table 1; EIMS *m*/*z* (rel. int.): 276(67), 260(50), 168(70), 167(100), 153(22) and 69(44).

*Mangostanin* (**3**). Yellow amorphous powder. Spectral data are in agreement with the literature [18].

*Mangostenol* (**4**). Yellow solid; m.p. 159–160 °C. Spectral data are in agreement with the literature [19].

## 4. Conclusions

A new prenylated xanthone, mangaxanthone B and a new benzophenone, mangaphenone, were isolated along with two known xanthones, mangostanin and mangostenol, from the stem bark of *Garcinia mangostana*. Biological evaluation of these compounds is under way.
